# Supplementary material for: Efficacy and Durability of Dolutegravir- or Darunavir-Based Regimens in ART-Naïve AIDS- or Late-Presenting HIV-Infected Patients
Source: Viruses. 2023 May 8;15(5):1123. doi: 10.3390/v15051123 (PMC10224150; doi:10.3390/v15051123)
Supplement: Supplementary file 1 [file viruses-15-01123-s001.zip › viruses-2347318-supplementary.pdf]

Supplementary Table S1. Description of AIDS events (n=187) in AIDS-presenter patients (n=124).

	N (%)
**Pneumocistis jirovecii pneumonia**	52 (28.8)
**Oesophageal candidiadis**	31 (16.6)
**CMV disease**	28 (15.0)
**Kaposi sarcoma**	18 (9.6)
**Wasting syndrome**	15 (8.0)
**Neurotoxoplasmosis**	9 (4.8)
**HIV-associated dementia**	9 (4.8)
**Non Hodgkin Lymphoma**	5 (2.7)
**Lung tuberculosis**	3 (1.6)
**Recurrent pneumonia**	3 (1.6)
**Cryptosporidiosis**	3 (1.6)
**Progressive multifocal leukoencephalopathy**	3 (1.6)
**Non tubercular mycobacterial disease**	3 (1.6)
**Histoplasmosis**	1 (0.5)
**Recurrent Salmonella spp sepsis**	1 (0.5)
**Disseminated HSV infection**	1 (0.5)
**Cryptococcal meningitis**	1 (0.5)
**Uterine cervical cancer**	1 (0.5)

Notes: 46 (37.1%) patients had ≥1 AIDS-event.
